# Patients Presented With Common Neurological Diseases Admitted to Referral Neuroscience Hospital in Bangladesh

**DOI:** 10.7759/cureus.90274

**Published:** 2025-08-17

**Authors:** Tahmina Dola, Alif Al Mamun, Sumaia Siddika, Md. Abdullah Yusuf

**Affiliations:** 1 Biotechnology, National Institute of Neurosciences and Hospital, Dhaka, BGD; 2 Biomedical Engineering, Australian National University (ANU), Sydney, AUS; 3 Neuroscience, National Institute of Neurosciences and Hospital, Dhaka, BGD; 4 Microbiology, National Institute of Neurosciences and Hospital, Dhaka, BGD

**Keywords:** ais (acute ischemic stroke), common neurological diseases, disability-adjusted life-years, global burden of diseases, sah- subarachnoid hemorrhage

## Abstract

Background

Neurological disorders have become a global disease burden and a major cause of morbidity and mortality.

Objective

The objective of the study was to identify the most common neurological diseases at Referral Neuroscience Hospital in Bangladesh.

Methodology

It is a retrospective study. It was conducted at the Referral Neuroscience Hospital in Bangladesh with the assistance of the neurology department. The study was carried out from January to June 2024. Data from the past five years were documented in this study. All the required data were obtained from the National Neuroscience Hospital’s yearbook covering 2018 to 2022. Information on the number of cases, their gender, type of disease, and the age of the patients was gathered and analyzed in Microsoft Excel (Redmond, USA).

Results

According to our investigation, stroke is seen to be the most common neurological disease among the top 10. It accounted for 26,971 patients. Intracerebral brain hemorrhage (ICH) had the highest number of patients (14169 cases), while acute ischemic stroke (AIS) and subarachnoid hemorrhage (SAH) had 8997 and 3805 patients, respectively. The number of patients for the other diseases was lower than two thousand. The third disease with the highest number of patients after stroke is Guillain-Barré syndrome (GBS), with 1625 cases. Meningoencephalitis has 1484 cases. The remaining diseases have less than a thousand cases. Cerebral venous sinus thrombosis (CVST) has 671 patients, and idiopathic intracranial hypertension (IIH) has almost half of that number (377 cases). Metabolic encephalitis and central nervous system tuberculosis (CNS TB) have nearly the same number of patients, with 592 and 575 cases, respectively. Peripheral neuropathy has the fewest patients at 295. Moreover, the number of male patients outnumbered female patients, with over 50.0% of patients being male for most diseases. During the years from 2018 to 2022, it is clear that the number of patients increased exponentially (2018: 4810 cases, 2019: 5428 cases, 2020: 6684 cases, and 2021: 8319 cases). However, in 2022, the number of patients was lower than the previous year (7201 cases) but still greater than in the rest of the years. The majority of patients were aged between 41 and 60.

Conclusion

In conclusion, stroke is seen to be the leading ailment, comprising almost 82% of the cases, whereas all the other seven diseases only held 18% of the cases. Regarding the male-female ratio of the cases, male patients were higher in number than female patients. The ages of the patients who were most admitted to the hospital were between 41 and 60. Moreover, the pre-COVID period (2018 and 2019) had a lower number of cases than the COVID period (2020 and 2021).

## Introduction

Neurological diseases cause disability and are the second most common cause of death around the world [1]. As a result, it has become an undeniable global public health challenge that will probably increase further in the next few decades [2]. Mechanistically, these disorders can undermine cognitive and motor function through white matter damage, abnormal amyloid deposition, blood-brain barrier disruption, synaptic plasticity damage, and impaired nerve conduction [3]. Recognizing that the brain serves as the command center for the human nervous system, it's crucial to acknowledge that any harm to the brain can be a matter of life and death. Even with the awareness that is leading to reduced fatality rates, some of the neurological disorders can still inflict long-lasting disabilities and suffering [4]. With the growth of the population and people living longer, more individuals are reaching ages when they are vulnerable to various types of neurological diseases. Thus, it leads to a global disease burden. Among neurological diseases, stroke is the primary cause of most disabilities and mortalities. As a result, it increases the economic burden and leads to more suffering [3].

For the past three decades, there has been an increase in the number of deaths (around 39%) following the disability-adjusted life-years (DALYs), accompanied by a 15% rise notwithstanding reductions in communicable neurological disorders. This scenario is mostly seen in middle-income and low-income countries (LMICs) [2]. The escalation of neurological disorders is primarily attributed to global population expansion and aging. Comprehending this burden has been further complicated by rapid shifts in demographic characteristics and risk factors such as overweight and obesity in both high-income countries (HICs) and LMICs [2]. To understand the underlying reason for the increase in the number of neurological diseases, cross-country and cross-region comparisons are essential. Moreover, it will also create a better understanding of the diseases’ diverse distribution, impact, and progression. The Global Burden of Diseases, Injuries, and Risk Factors Study (GBD) 2016 emphasized the importance of the study of neurological diseases such as stroke, migraine, Alzheimer’s disease, and other dementias. Their comprehensive study thoroughly evaluated the global, regional, and national burden of diseases [3].

In Bangladesh, neurology was established as one of the medical specialties during the 1960s. The incidence of stroke is at a steady rise. Bangladesh is a developing country, and the average life expectancy is 60 years. Surveys show that infectious diseases and malnutrition contribute to about one-fifth of the 43 million deaths per year. A significant number of people also suffer from early-onset cerebrovascular disease, where stroke carries a higher risk of mortality. Along with the suffering, many people cannot receive the correct and necessary treatment in Bangladesh [5]. On top of that, the social and cultural stigma surrounding neurological diseases is one of the reasons that people do not get good treatment [6]. Moreover, a major portion of the people reside in rural areas where they do not have adequate facilities to manage these types of incidents. All these problems lead to neurological disease, a significant health concern in this country, impacting individuals of all genders and ages. While this disease is mostly seen in elderly people, younger people are also facing the same problems and diseases. This study aims to identify and analyze the most common neurological diseases treated at the Referral Neurosciences Institute in Bangladesh, documented in the Yearbook of the National Institute of Neurological Diseases and Hospital.

## Materials and methods

Study settings and population

This is a retrospective study covering the data collected from the Yearbook of the National Institute of Neurosciences & Hospital. People from all around Bangladesh come to this hospital for treatment. It is a government hospital, inaugurated on 12th September 2012, where people get treatment at a lower price. People of all ages can get treatment from this hospital. Neurological diseases like stroke, epilepsy, Guillain-Barré syndrome (GBS), cerebral venous sinus thrombosis (CVST), migraine, Alzheimer's disease, etc., are treated. All relevant information regarding the admitted patients was obtained from each neurology department's records and approved by the unit's head. During the data collection, the personal information of the patients was kept confidential, keeping them private. The data covered the years 2018 to 2022. The data comprises the number of patients, the top 10 neurological diseases, the number of male and female patients, and their ages, pre-COVID and COVID period scenarios, etc. This study was performed for six months (from January 2024 to July 2024). Any unnecessary and incomplete information was excluded from the analysis.

Study procedure

The data was collected from the annual yearbook of the National Institute of Neurosciences and Hospital. This yearbook is published annually and is found on the hospital’s official website [7]. This publication provides news and data that are found in patients’ profiles across all six units within the neurology department. The profile includes the type of neurological disease the patient has and the demographic information, including gender, age, locality, mortality, and morbidity. The yearbook data may include new and recurrent CVA cases. The data required for this analysis were obtained from annual publications over the past five years. Following the acquisition of data for each year, we discerned the top ten neurological diseases from that period. Additionally, we compiled information regarding the age and gender distribution related to these diseases.

Statistical analysis

The data required for the study analysis were organized in an Excel spreadsheet. The data were collected from the yearbooks. After the data organization, it was ready for analysis. The dataset covered all the necessary information-the diseases, along with the number of cases of each of the diseases, gender, age, and prevalence during the pre-COVID and COVID periods. After data entry, Excel sheets were utilized for calculation. All these data enabled our comprehensive and detailed analysis of the neurological disease landscape within the studied population.

Ethical clearance

Ethical approval was received from the Institutional Review Board (IRB) of the referral hospital, the National Institute of Neurosciences and Hospital, for this study. The data were not disclosed, and the patients’ privacy was maintained. The necessary data obtained during the study were kept confidential and were used solely for the study. All methods were carried out according to the hospital guidelines and regulations.

## Results

In this article, we accumulated data on the diagnosed diseases, which were documented in the yearbooks from 2018 to 2022. First, we listed all the diagnosed diseases that had the largest number of cases. This list was done on the top 10 neurological diseases. This was done for each year and all six units. As a result, we were able to document the top 10 neurological diseases from each year and each unit. This approach resulted in the documentation of 30 diseases over the five years. The top ten neurological diseases that are presented in this study were determined by calculating the total number of cases for each of the diseases. The top 10 neurological diseases over the 5 years were intracerebral brain hemorrhage (ICH), acute ischemic stroke (AIS), subarachnoid hemorrhage (SAH), Guillain-Barré syndrome (GBS), meningoencephalitis, CNS TB, metabolic encephalitis, cerebral venous sinus thrombosis (CVST), idiopathic intracranial hypertension (IIH), and peripheral neuropathy.

**Figure 1:**
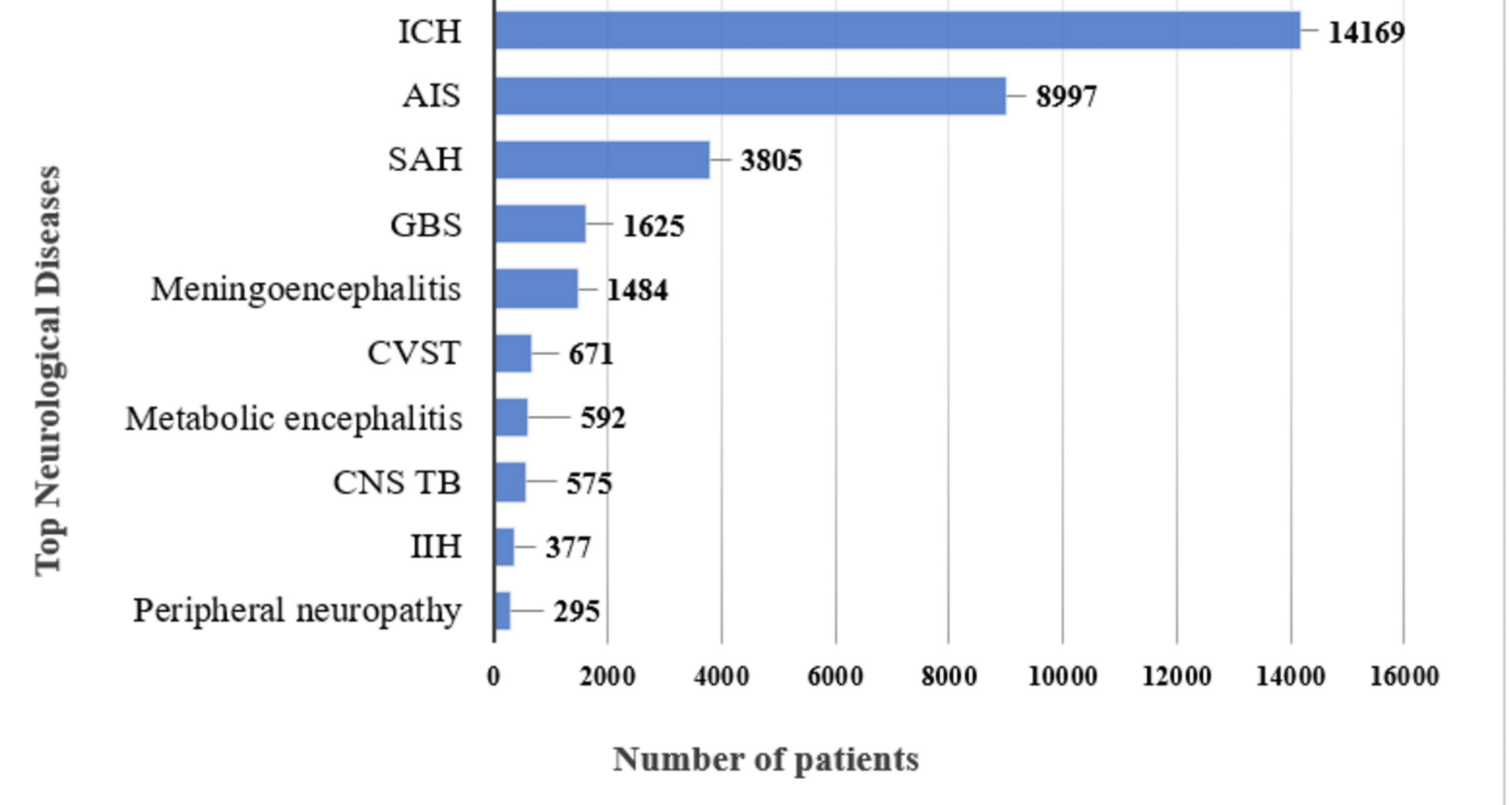
Top ten neurological diseases and their prevalence. The image is an analysis by the authors based on the data collected from the Yearbook (2018–2022) and the National Institute of Neurosciences and Hospital. ICH: Intracerebral hemorrhage, AIS: Acute ischemic stroke, SAH: Subarachnoid hemorrhage, GBS: Guillain-Barré syndrome, CNS TB: Central nervous system tuberculosis, IIH: Idiopathic intracranial hypertension, CVST: Cerebral venous sinus thrombosis

The data presented in Figure 1 is a demonstration of the data obtained from the yearbook. It illustrates the prevalence of the top 10 neurological diseases and the number of patients. The graph shows that ICH had the highest number of cases (14169 cases) compared to all other diseases, while peripheral neuropathy is at the bottom, as it has the lowest number of patients (295 cases). The patient count for ICH exceeded that of AIS (8997 cases) by nearly one and a half times. Conversely, the patient count for AIS surpassed that of subarachnoid hemorrhage (SAH) by almost double, with SAH having 3805 cases. Additionally, the patient counts for Guillain-Barré syndrome (GBS) (1625 cases) and Meningoencephalitis (1484 cases) were approximately equal, as were the counts for metabolic encephalitis (592 cases) and CNS TB (757 cases). IIH had 377 cases, and the remaining diseases each had fewer than 700 cases. In the yearbook that covers the period from 2018 to 2022, all diagnosed diseases were included, but only the top 10 neurological diseases were extracted from the dataset for each year. The top 10 neurological diseases over the five years were determined based on the total number of patients.

**Figure 2:**
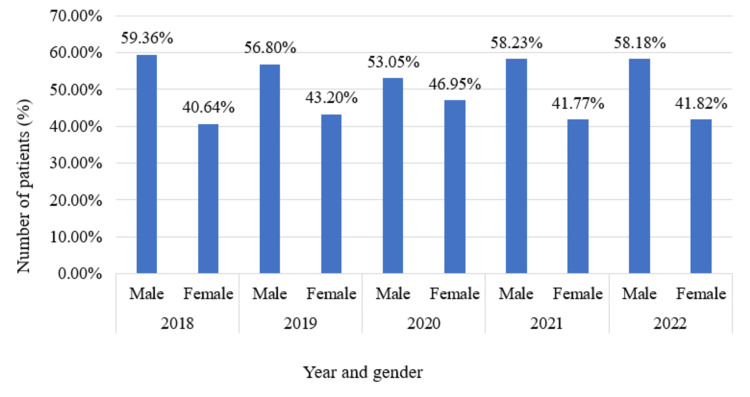
Number of male and female patients from 2018 to 2022. The image is an analysis by the authors based on the data collected from the Yearbook (2018–2022) and the National Institute of Neurosciences and Hospital.

Figure 2 depicts that the number of male patients was higher than the number of female patients every year. Throughout the years, the proportion of male patients was consistently above 53%. However, there was a decrease in the total number of cases from 2018 to 2019. In 2018, the percentage of male patients was highest (59.36%), while the percentage of female patients was lowest (40.64%). However, for the rest of the years, the number of female patients was higher. In 2020, the proportion of male patients was at its lowest (53.05%), while the proportion of female patients was at its highest (46.95%). The fraction between male and female patients was nearly equal in 2021 and 2022 (males more than 58% and females more than 41%).

**Figure 3:**
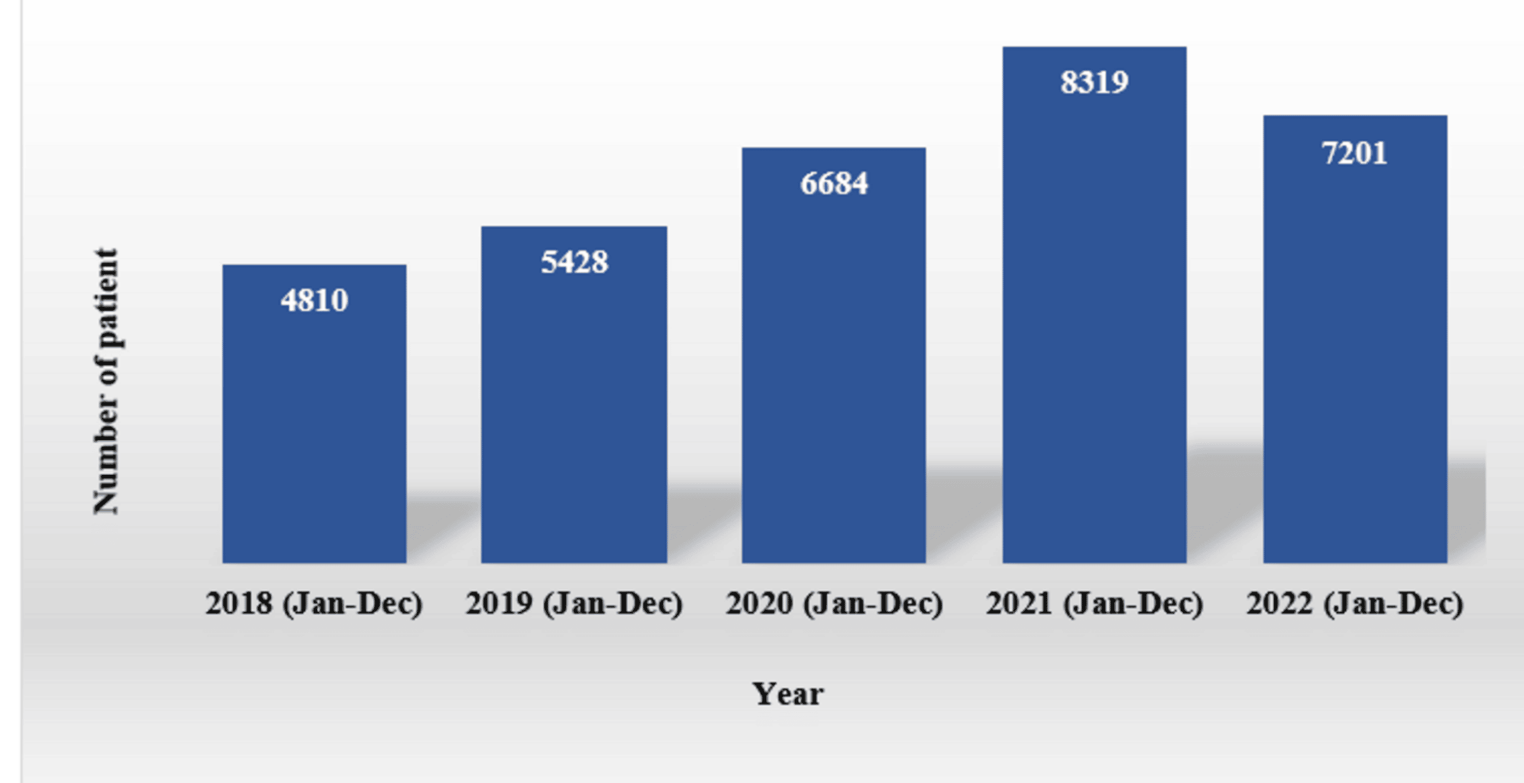
Number of total patients from 2018 to 2022. The image is based on the authors’ analysis of data collected from the Yearbook (2018–2022) and the National Institute of Neurosciences and Hospital.

In reviewing the total number of patients for each year (Figure 3), it is evident that patient volumes had an incremental growth from 2018 to 2021. Specifically, there was an increase of one thousand patients from 2019 to 2020 and a subsequent increase of two thousand from 2020 to 2021. In 2022, the number of cases decreased relative to 2021. However, the number remained higher than in previous years. The years 2018 and 2019 denote the pre-COVID period, while the subsequent period pertains to the time during COVID-19. The number of cases was already increasing at a steady rate, but the number during the COVID period was higher than in the pre-COVID period. Considering the pre-COVID and COVID periods as two variables, p>0.05 was considered statistically significant. Here, the p-value is 0.17>0.05. It indicates that the result is not statistically significant.

The years 2018 and 2019 denote the pre-COVID period, while the subsequent period pertains to the time during COVID-19. The number of cases was already increasing at a steady rate, but the number during the COVID period was higher than in the pre-COVID period. Considering the pre-COVID and COVID periods as two variables, p>0.05 was considered statistically significant. Here, the p-value is 0.17>0.05. It indicates that the result is not statistically significant. The data is presented in Table 1.

**Table 1:** Change in patient numbers before and during the COVID-19 pandemic. The p-value here was calculated by Welch’s t-test.

Pre-COVID		COVID-period		p-value	t-test
Year	Number of patients	Year	Number of patients		
2018	4810	2020	6684	0.22	-2.72
2019	5428	2021	8319		

Table 2 represents the number of cases in each year. Each bar contains one particular disease and the five years (vertically). It is evident that in the years 2021 and 2022, the number of cases drastically increased compared to any other year.

**Table 2:** The number of cases of the top neurological diseases from 2018 to 2022. ICH: Intracerebral hemorrhage, AIS: Acute ischemic stroke, SAH: Subarachnoid hemorrhage, GBS: Guillain-Barré syndrome, CNS TB: Central nervous system tuberculosis, IIH: Idiopathic intracranial hypertension, CVST: Cerebral venous sinus thrombosis

Year	ICH	AIS	SAH	GBS	Meningo- encephalitis	CVST	Metabolic- encephalitis	CNS TB	IIH	Peripheral neuropathy	Total
2018	2415	1279	565	26	192	25	101	130	46	31	4810
2019	2253	1620	701	272	241	112	72	44	113	0	5428
2020	2874	1829	676	309	323	228	168	123	64	90	6684
2021	3930	2380	754	367	300	175	166	92	37	118	8319
2022	2697	1949	1109	428	429	148	100	152	133	56	7201
Total	14169	9057	3805	1402	1485	688	607	541	393	295	32442

**Figure 4:**
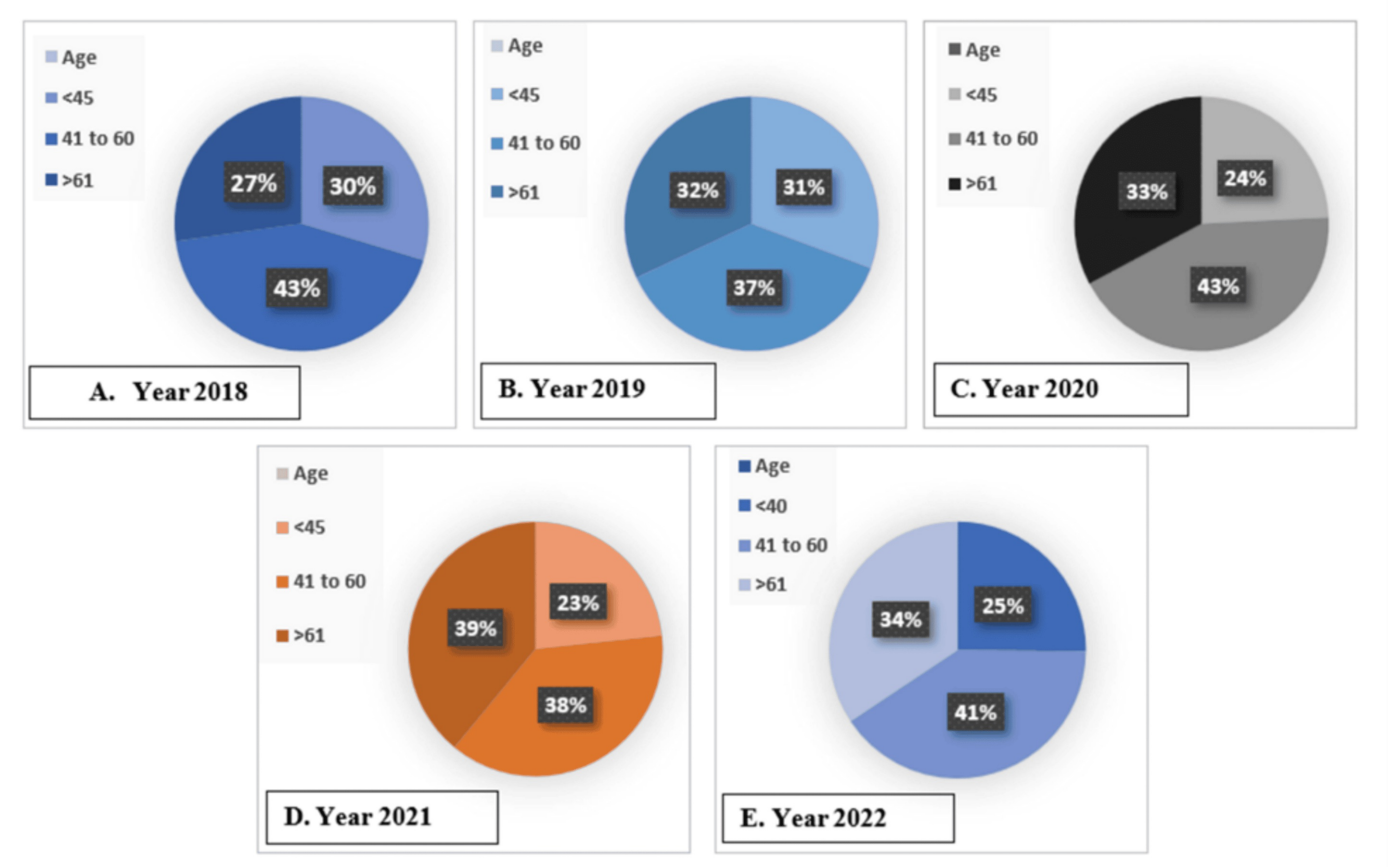
The number of patients aged <45, 41-60, and >61. A. year 2018, B. year 2019, C. year 2020, D. year 2021, E. year 2022. The image is based on the authors’ analysis of the data collected from the Yearbook (2018–2022) and the National Institute of Neurosciences and Hospital.

Age was one of the variables that we included in our study. The result of Figure 4 demonstrates that over the years, people aged 41 to 60 had the highest number of cases of neurological diseases. It comprises almost 35% to 45% of the total number. However, in 2021, there was a small dissimilarity, as the number of cases increased above 60. It was around 39%, whereas people aged 41 to 60 were 38%. All these data points point to further investigation for a better understanding and finding the cause of the increased number of cases.

## Discussion

Neurological disorders pose a significant burden of disease worldwide, and the spectrum of diseases ranges from stroke and neurodegenerative disorders to central nervous system infections. The worldwide prevalence of neurological disorders is high [5]. Keeping that in mind, this study was conducted to see the occurrence of neurological diseases and to identify the top 10 neurological diseases recorded in the National Institute of Neurosciences & Hospital. In our study, we separated intracerebral hemorrhage (ICH) and subarachnoid hemorrhage (SAH), as they were recorded in distinct diagnostic categories in the yearbooks. 

In various investigations, it has been presented that intracerebral hemorrhagic stroke is less prevalent than acute ischemic stroke. Sharif et al. showed in their investigation that AIS accounted for approximately 75% of all strokes, while hemorrhagic stroke accounted for 25% [8]. Similarly, Schaub et al. also reported that the prevalence of AIS is greater than that of ICH [9]. However, in our dataset, intracerebral hemorrhage (ICH) has the largest number of patients, followed by acute ischemic stroke (AIS) and subarachnoid hemorrhage (SAH) (ICH-14169, AIS-8997, SAH-3805). This dissimilarity may be explained by the fact that the National Institute of Neurosciences and Hospital is a specialized tertiary referral center in Bangladesh, which receives a disproportionately high number of severe hemorrhagic stroke cases. Moreover, this pattern can also be influenced by referral bias, variation in case definitions, and underreporting of ischemic cases. Research by Chowdhury et al. [6] and Shahab Uddin et al. [10] has also consistently demonstrated the predominance of these three types of strokes (ICH, AIS, and SAH) over other diseases. A report highlights stroke as a significant contributor to mortality and disability in the region, with an age- and sex-standardized mortality [11]. In Bangladesh, the rate of stroke is 54.8 per 100,000, with 888.1 disability-adjusted life years lost per 100,000 [12], and in the United States, stroke is the fifth leading cause of death, resulting in the deaths of nearly 129,000 people annually, which is considered the leading cause of long-term severe disability [10]. These statistics underscore the prominence of stroke as the most prevalent neurological condition.

In our research, we have found that there are more male patients than female patients. There may be several factors contributing to this difference. The demographic profile of the patients in this study reflects that neurological disorders were more prevalent among males (57.19%). One main reason for the sex-related disparity in stroke cases could be the variation in sex steroid hormones. Estrogen in females promotes vasodilation that improves blood flow. It offers some protection against ischemic damage. On the other hand, testosterone causes vasoconstriction, causing reduced blood flow, potentially increasing the stroke risk [13]. This hormonal layout could be responsible for more male stroke cases than female cases. Genetic and anatomical factors, in conjunction with lifestyle choices such as levels of physical activity, dietary habits, social interactions, and cigarette smoking, may collectively contribute to variations in stroke epidemiology, pathophysiology, and clinical outcomes according to gender. It is crucial to keep in mind that alcohol and cigarettes have a link with neurological diseases [12]. In Bangladesh, the consumption of alcohol and smoking among women is less likely than among men; as a result, it might be an important factor in saying that it could be one of the reasons responsible for a higher number of cases of neurological diseases among men than women. Sex may have another potentially exerted influence on the disease process through disparities in chromosomal complement, gene expression, hormonal profiles, organ systems, and a spectrum of physiological processes [14].

Age is a significant factor in neurological diseases. Our data reveals that individuals aged 41 to 60 are more vulnerable to these disorders. However, it is also seen that people under 41 and over 60 are being affected. Based on the research by Chowdhury et al. and our research, it is evident that there is a higher number of cases between the ages of 41 and 60 compared to other age groups [6]. The prevalence of disabling neurological disorders rises notably with age. As our population ages, particularly in low-income countries, our societies will encounter increasing challenges in providing treatments, rehabilitation, and addressing the consequences of these neurological disorders [15].

The main indication of a COVID-19 infection is pneumonia. Research has shown that viruses have the ability to affect organs as well as the central and peripheral nervous systems. Studies on coronaviruses stated that they can affect the nervous system [16,17]. The complexity of the nervous system may result from the direct action of the viruses on the nervous tissues or by compromising the mechanism of the immune system. The impact on the nervous system can be observed during the acute phase of the disease as well as days, weeks, or months following the acute phase. An investigation from Wuhan, China, revealed that 78 out of 214 cases (36.4%) with COVID-19 infection exhibited neurological manifestations. These fell into three categories: central nervous system, peripheral nervous system, and musculoskeletal symptoms [18]. Patients with severe infection experienced stroke, ataxia, seizures, and depressed levels of consciousness. A retrospective study in Wuhan found that 11 out of 221 COVID-19 patients developed acute ischemic stroke, one had cerebral venous sinus thrombosis, and one suffered cerebral hemorrhage. However, patients with these severe complications were more likely to be elderly with medical comorbidities, especially vascular risk factors such as hypertension [19].

Apart from coronavirus, there are other viruses that are associated with neurological complications. An interesting investigation claimed that the swine flu vaccine led to increased cases of GBS and other infectious or immune-mediated diseases [20]. Several viral infections can lead to damage to the nervous system, which causes encephalitis, toxic encephalopathy, and post-infectious demyelinating diseases [21]. It was reported that it had attacked the nervous system during the 1919 pandemic [22]. The US government asked Leonard T. Kurland, a prominent US neuroepidemiologist from the Mayo Clinic in Rochester, Minnesota, USA, to collect and analyze data on the incidence of several infectious and immune-mediated inflammatory neurological diseases [23-26] along with the investigation of the people who had been vaccinated and had similar symptoms because of the vaccines. This incident caused the US government to make a detailed investigation into the connection between vaccines and neurological diseases.

Upon scrutinizing the annual tally of patients with the top neurological diseases, a striking surge in cases, particularly strokes, was evident during the pandemic compared to the previous two years. Given that hypertension, anxiety, and stress can trigger strokes, it is plausible that the pandemic-induced anxiety and distress among the populace are fueling the spike in stroke cases. Although the number had been gradually increasing in previous years, the number increased significantly in 2020 and 2021. The number slightly decreased the following year in 2022, but the number was still greater than in the years 2018 and 2019, as evidenced by Table 2.

In Bangladesh, a significant proportion of individuals suffer from prevalent neurological disorders and are unable to access adequate treatments. It is not only because of the economic strains but also because of the unawareness of the general people. The field of epidemiology serves a critical role in understanding the patterns and prevalence of diseases occurring within the community. Unfortunately, our understanding and access to the prevalence and frequency of neurological diseases within our specific local context are currently inadequate [10].

Strength of the study

This study mainly sought to look for the top ten neurological diseases in Bangladesh. Also, we tried to figure out which year had the most cases, whether the pandemic had any effect, and the age group of people who were mostly diagnosed with neurological diseases.

Limitations

As it is a retrospective study, it was challenging to avoid prejudices. Also, this study has only covered the past five years, 2018 to 2022. The data of the current study were extracted from the hospital documents. As a result, the study subjects and their caregivers were not directly involved in the study design and protocols. Additionally, the history and risk factors of the patients were not included due to data source limitations.

## Conclusions

Worldwide, the number of patients facing neurological diseases is increasing, and Bangladesh is not exceptional, as evidenced in hospital yearbooks. Notably, strokes were the most severe of all the diseases. Along with stroke, there are other neurological diseases that are listed as top neurological diseases. They are Guillain-Barré syndrome (GBS), meningoencephalitis, CNS TB, metabolic encephalitis, cerebral venous sinus thrombosis (CVST), Idiopathic intracranial hypertension (IIH), and peripheral neuropathy. Our data showed that the number of male patients is higher than that of female patients. Physical conditions and lifestyles could be the primary reasons behind the larger number of male patients. Other conditions like age, chronic diseases, unhealthy lifestyle, etc., can be the root cause of these illnesses. One of the major concerns is that the hospital's location is in the capital, which results in undocumented cases. Overall, making people aware of the current condition, leading a healthy lifestyle, cultivating gender equality, and enhancing medical treatments and technology can mitigate the severity of neurological diseases.

## Appendices

Acknowledgement

We are thankful to the record staff and the doctors of the National Institute of Neurosciences and Hospital who permitted us to contact documents of the admitted patients. A special thanks to the data collectors and all the study subjects whose information was used for the present study.

Author contributions

The authors of the study made a significant contribution throughout the study, in the conception, study design, execution, data analysis, and interpretation; they took part in drafting, revising, and critically reviewing the article. Lastly, they gave their approval for the publication.
